# LncRNA PCGEM1 accelerates non-small cell lung cancer progression via sponging miR-433-3p to upregulate WTAP

**DOI:** 10.1186/s12890-020-01240-5

**Published:** 2020-08-12

**Authors:** Lei Weng, Kejie Qiu, Weijing Gao, Chunbo Shi, Fen Shu

**Affiliations:** Department of Respiratory and Critical Care Medicine, Ningbo Ninth Hospital, No.68 Xiangbei Road, Jiangbei District, Ningbo, 315020 Zhejiang China

**Keywords:** PCGEM1, miR-433-3p, WTAP, Non-small cell lung cancer

## Abstract

**Background:**

Non-small cell lung cancer (NSCLC) is one of the most common malignant tumors all over the world. In recent years, long non-coding RNAs (lncRNAs) have been proven to participate in the development of different cancers, including NSCLC. PCGEM1 prostate-specific transcript (PCGEM1) is the lncRNA which is associated with the progression of several cancers. Nevertheless, in NSCLC, the specific functions of PCGEM1 are not yet clear.

**Methods:**

The real-time quantitative polymerase chain reaction (qPCR) was utilized to test the expression of PCGEM1 in NSCLC cells. Functional experiments, including cell Counting Kit-8 (CCK-8) assay, 5-ethynyl-2’-deoxyuridine (EdU) assay, flow cytometry analysis and transwell assays were utilized to estimate cell proliferation, migration, invasion and apoptosis. Meanwhile, RNA pull down assay and luciferase reporter assay were utilized to evaluate the correlation of miR-433-3p with PCGEM1 or WT1 associated protein (WTAP).

**Result:**

PCGEM1 was highly expressed in NSCLC cells, while miR-433-3p was lowly expressed in NSCLC cells. PCGEM1 silencing or miR-433-3p overexpression inhibited cell proliferation, migration and invasion but accelerated cell apoptosis. MiR-433-3p was proven be sponged by PCGEM1. Besides, WTAP was the target of miR-433-3p and it accelerated the progression of NSCLC. In the end, rescue experiments indicated that overexpression of WTAP or knockdown of miR-433-3p reversed the inhibited roles of silencing PCGEM1 on cell behavior.

**Conclusions:**

PCGEM1 accelerates NSCLC progression via sponging miR-433-3p to upregulate WTAP.

## Background

Non-small cell lung cancer (NSCLC) is one of the most common malignant tumors all over the world. In recent years, it has become the first cause of cancer-related death in urban population [1]. NSCLC accounts for about 80% in the whole lung cancer cases [2]. Moreover, because the immature technology of early screening, the vast majority of patients have reached the advanced stage when they are diagnosed [3]. With the development of medicine, great progress has been made in the treatment strategies. However, the prognosis of patients is still unsatisfactory and the five-year survival rate is less than 20% [4]. Therefore, it is extremely crucial to discover effective biomarkers for the treatment of NSCLC.

Long non-coding RNAs (LncRNAs) are transcripts without the protein-coding capability. They consist of more than 200 nucleotides in length [5]. LncRNAs are associated with numerous biologic processes, such as cell proliferation, migration and apoptosis [6, 7]. In recent years, an increasing number of reports indicate that lncRNAs are frequently to be abnormally expressed in assorted cancers [8]. They exert the functions of tumor suppressor or facilitator in the progression of human cancers. For example, SPRY4-IT1 can sponge miR-101-3p to expedite cell proliferation and invasion of bladder cancer via regulating EZH2 [9]. NEAT1 exerts the tumor-accelerating functions in esophageal squamous cell carcinoma through miR-129/CTBP2 axis [10]. CPS1-IT1 has been confirmed to suppress the proliferative and migrated capabilities of colorectal cancer cells [11]. PCGEM1 prostate-specific transcript (PCGEM1) is an lncRNA which has been searched in several cancer types. For example, PCGEM1 is highly expressed in prostate cancer cells, and it can accelerate the progression of prostate cancer through sponging miR-145 [12]. It has also been proven that PCGEM1 can affect the cellular processes in endometrial carcinoma via sponging miR-129-5p and regulating STAT3 [13]. Moreover, overexpressed PCGEM1 can expedite cell proliferation in ovarian carcinoma via RhoA pathway [14]. Nevertheless, there are few researches on the functions of PCGEM1 in NSCLC, and the specific regulatory mechanisms of PCGEM1 are also unclear.

In recent years, a plenty of researches have confirmed that competing endogenous RNA (ceRNA) network exerts the crucial role in human cancers. In a ceRNA network, lncRNA can act as a sponge of microRNAs (miRNAs) to release messenger RNA (mRNA) so as to regulate cancer progression [15]. For example, lncRNA OGFRP1 upregulates LYPD3 expression by sponging miR-124-3p and promotes NSCLC progression [16]. PCGEM1 has also been proven to promote cell proliferation, migration and invasion via targeting the miR-182/FBXW11 axis in cervical cancer [17]. However, whether PCGEM1 can function as a ceRNA to regulate NSCLC progression is still unclear.

The fundamental purpose of the current research was to investigate the functions and mechanisms of PCGEM1 in NSCLC, thereby offering the new insight to make the investigation in the therapeutic schedule of NSCLC.

## Methods

### Clinical specimens

NSCLC and adjacent normal lung tissues used in this study were obtained from 50 patients who underwent primary surgical resection in Ningbo Ninth Hospital. No patients received any kind of treatment before operation. As soon as collection, the tissue samples were immediately frozen in liquid nitrogen and stored at − 80 °C for following experiments. The protocol of the current study was approved by Ningbo Ninth Hospital, and all participants had provided the informed consent form.

### Cell culture

Human lung bronchial epithelial cell line (BEAS-2B) and human non-small cell lung cancer cell lines (A549, NCI-H1299, NCI-H1650) were all available from ATCC (Manassas, VA, USA) and maintained at 37 °C in an incubator supplied with 5% CO_2_. Human non-small cell lung cancer cell line PC-9 was purchased from COBIOER Company (Nanjing, China). BEAS-2B cell line was cultivated in BEGM medium (Gibco) with LHC-9 media (Gibco, USA). F-12 K medium (Gibco) was utilized to cultivate A549 cells with 10% FBS. NCI-H1299, NCI-H1650 and PC-9 cells were routinely cultured with 10% FBS in RPMI-1640 medium (Gibco).

### Total RNA isolation, reverse transcription and quantitative real-time polymerase chain reaction (qPCR)

Isolation of total RNA was performed with application of TRIzol Reagent (Invitrogen, Carlsbad CA, USA), and cDNA template was acquired via PrimeScript Reverse Transcriptase Kit (Takara, Shiga, Japan) for qPCR. The SYBR Green PCR Kit (Takara) was commercially obtained and used on Step-One Plus Real-Time PCR System (Applied Biosystems, Foster City, CA, USA). Results were processed by 2^−ΔΔCt^ method, using GAPDH or U6 as normalized gene.

### Plasmid transfection

The specific short hairpin RNAs (shRNAs; GenePharma, Shanghai, China) to PCGEM1 and WTAP were applied for silencing expressions of PCGEM1 and WTAP, by utilizing nonspecific shRNAs as negative control (NC). The pcDNA3.1 vector (Invitrogen) containing the full-length of WTAP cDNA, was used to overexpress the expression of WTAP, with empty pcDNA3.1 vector as NC. The miR-433-3p inhibitor, miR-433-3p mimics and NCs were procured from Ribobio (Guangzhou, China). NCI-H1299 and NCI-H1650 cells were harvested for 48 h of transfection in 6-well plates, with application of Lipofectamine 3000 (Invitrogen). Bio-repeats were run in triplicate.

### 5-ethynyl-2’-deoxyuridine (EdU) assay

For EdU staining assay, the transfected cells (1 × 10^4^) of NCI-H1299 and NCI-H1650 were prepared in 96-well plates and cultivated with EdU kit (RiboBio) for 2 h at 37 °C. Following staining in DAPI solution for 5 min, cells were observed by use of fluorescence microscope (Olympus, Tokyo, Japan). Bio-repeats were run in triplicate.

### Cell counting kit-8 (CCK-8) assay

After transfection, NCI-H1299 and NCI-H1650 cells were reaped and seeded in 96-well plates with the cell density of 5 × 10^3^ cells in each well. 10 μL of CCK-8 solution purchased from Dojindo (Kumamoto, Japan) was added into plates after cells were incubated in several different time points. After that, the optical density values were examined at 450 nm with application of spectrophotometer (Thermo Fisher Scientific, Waltham, MA, USA). Bio-repeats were run in triplicate.

### Flow cytometry

1 × 10^6^ transfected cells (NCI-H1299 and NCI-H1650) were prepared and seeded in 6-well plates for cell apoptosis assay, in presence of Annexin V-FITC/PI double staining kit (Invitrogen). After 15 min of double-staining in the dark, cells were subjected to flow cytometer, as instructed by provider (BD Biosciences, Franklin Lakes, NJ, USA). Bio-repeats were run in triplicate.

### Transwell assay

Cell invasion or migration were examined with application of the 24-well transwell chambers (8-mm pore size; Corning Incorporated, Corning, NY, USA) coating with Matrigel (BD Biosciences) or not. After transfection, 2 × 10^4^ cells (NCI-H1299 and NCI-H1650) were reaped and added into upper chamber with serum-free medium. The lower chamber was added with 100% medium. After 24 h, cells on the bottom of membrane were fixed by 4% paraformaldehyde, and visualized under optical microscope (Olympus) after crystal violet staining. Five fields were randomly chosen. Bio-repeats were run in triplicate.

### Subcellular fractionation assay

Subcellular fractionation assay was undertaken in NCI-H1299 and NCI-H1650 cells with application of the PARIS™ Kit (Invitrogen) as guided. Cell cytoplasm and cell nucleus were separately isolated using cell fractionation buffer and cell disruption buffer. qRT-PCR was used to analyze the PCGEM1 content in cell fractions. Bio-repeats were run in triplicate.

### Luciferase reporter assay

The fragment of PCGEM1 or WTAP containing the wild type and mutant interacting sites of miR-433-3p, were prepared for conducting luciferase assays. Next, cells were co-transfected for 48 h with miR-433-3p mimics or NC mimics, and the pmirGLO vectors (Promega, Madison, WI, USA) which contained PCGEM1 or WTAP fragment. At length, luciferase assay was achieved with application of Dual-luciferase reporter assay system (Promega). Bio-repeats were run in triplicate.

### RNA pull down assay

RNA pull down assay in NCI-H1299 and NCI-H1650 cells was achieved in light of the protocol of Pierce Magnetic RNA-Protein Pull-Down Kit (Thermo Fisher Scientific). The wild type and mutant PCGEM1 or WTAP binding sites in miR-433-3p sequence were biotin-tagged into bio-miR-433-3p WT/MUT. Cell proteins were treated with the biotinylated probes and magnetic beads. The captured RNA-protein mixture was subjected to QRT-PCR. Bio-repeats were run in triplicate.

### Statistical analysis

All statistical analyses were completed by one-way ANOVA or Student’s t-test, employing GraphPad Prism 6.0 software (GraphPad, Inc., La Jolla, CA, USA). Bio-repeats were run in triplicate in each experiment, and experiment data were given as the means ± SD. Data were collected when *p*-value was below 0.05.

## Results

### PCGEM1 is highly expressed in NSCLC cells and knockdown of it suppresses cell growth

**Fig. 1:**
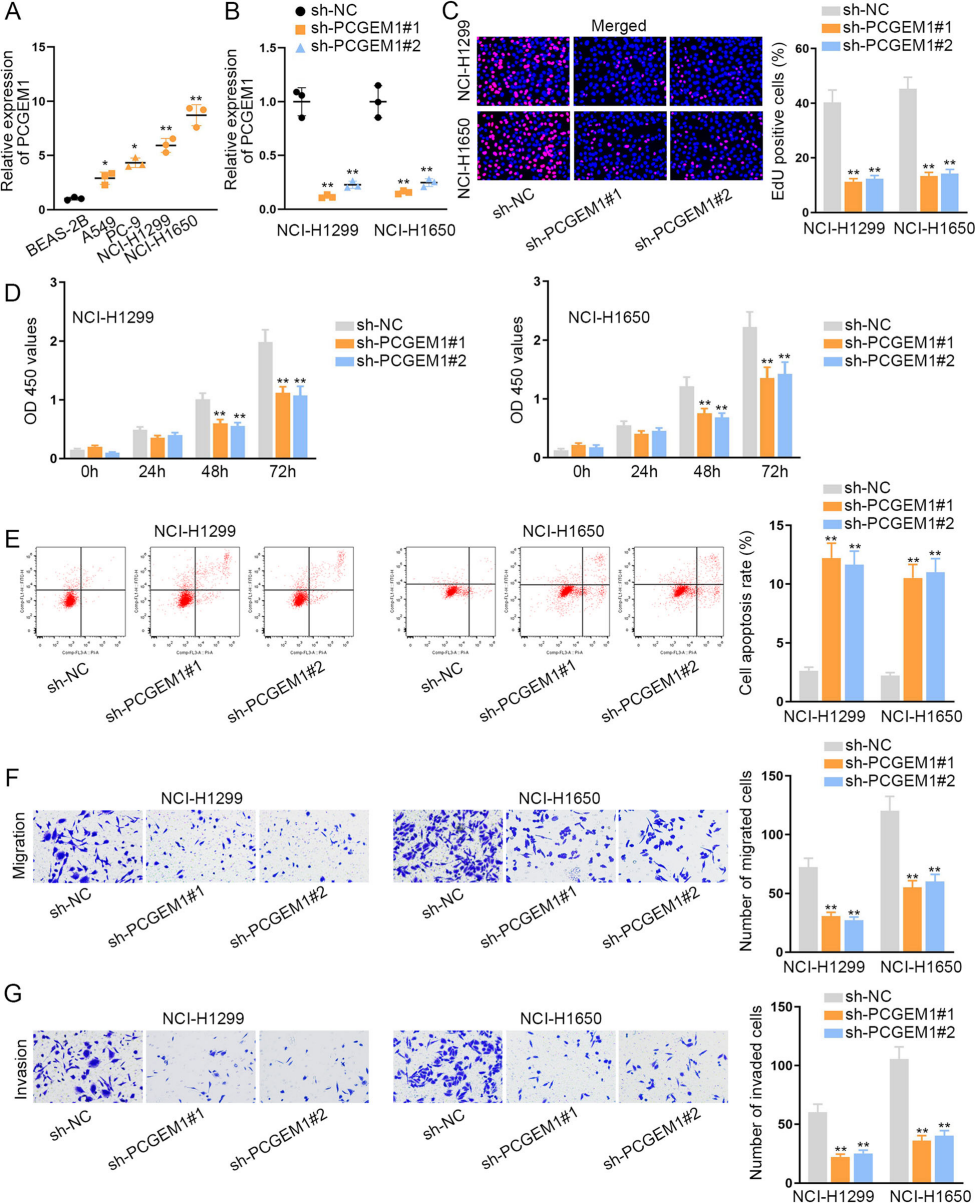
PCGEM1 is highly expressed in NSCLC cells and knockdown of it suppresses cell growth. **a** qRT-PCR was utilized to test the expression of PCGEM1 in NSCLC cell lines (A549, PC-9, NCI-H1299 and NCI-H1650) and the human lung bronchial epithelial cell line (BEAS-2B). **b** The interference efficiency of PCGEM1 in NCI-H1299 and NCI-H1650 cells was tested through qRT-PCR. **c**, **d** EdU and CCK-8 experiments were utilized to measure the cell proliferation when PCGEM1 was inhibited in NCI-H1299 and NCI-H1650 cells. **e** Flow cytometry analysis was implemented to evaluate cell apoptosis after PCGEM1 was knocked down in NCI-H1299 and NCI-H1650 cells. **f**, **g** Transwell assays were conducted to test cell migration and invasion after silencing of PCGEM1 in NCI-H1299 and NCI-H1650 cells. ^*^P < 0.05, ^**^P < 0.01

First of all, the expression level of PCGEM1 was examined. We utilized qRT-PCR analysis to test PCGEM1 expression in NSCLC tissues and cell lines. Intriguingly, we detected a relative high level of PCGEM1 in NSCLC tissues compared to adjacent normal tissues (Fig. S1A). Consistently, we discovered a notable high expression of PCGEM1 in NSCLC cell lines (A549, PC-9, NCI-H1299 and NCI-H1650) in contrast to the human lung bronchial epithelial cell line (BEAS-2B). As presented in Fig. 1a, the expression of PCGEM1 in NCI-H1299 and NCI-H1650 cells was the highest. Thus, we selected these two cells to conduct the further experiments. Next, we knocked down PCGEM1 expression in NCI-H1299 and NCI-H1650 cells separately by transfection with sh-PCGEM1#1/2. Then, qRT-PCR was utilized to test the interference efficiency of PCGEM1 (Fig. 1b). After that, we carried out loss-of-function experiments to evaluate the influence of PCGEM1 silencing on cell behaviors. With the conduction of EdU assay, we discovered that the ratio of EdU positive cells was decreased in NCI-H1299 and NCI-H1650 cells transfected with sh-PCGEM1, which indicated that cell proliferation was restrained by the depletion of PCGEM1 (Fig. 1c). Following, the results of CCK-8 assay further proved that cell proliferation could be repressed by PCGEM1 knockdown since the optical density (OD) 450 values were declined in PCGEM1-silenced NCI-H1299 and NCI-H1650 cells (Fig. 1d). In accordance with the flow cytometry analysis, the number of apoptotic cells were visibly ascended after PCGEM1 was inhibited in NCI-H1299 and NCI-H1650 cells, suggesting that cell apoptosis was facilitated by the silencing of PCGEM1 (Fig. 1e). Additionally, transwell experiments revealed that the numbers of migrated cells and invaded cells were reduced by the lack of PCGEM1, demonstrating that cell migrated and invaded capabilities were suppressed (Fig. 1f, g). TP53 involves in various biological processes. Considering the transcriptional regulatory role of p53, we detected the transcriptional activity of PCGEM1 in two NSCLC cells with p53 overexpression. As a result, p53 had no significant effect on PCGEM1 transcription (Fig. S1B). In addition, we analyzed whether PCGEM1 regulated p53 to exert functions. Through western blot analysis, we determined that PCGEM1 depletion had positive effect on the level of p53 protein (Fig. S1C). Moreover, the apoptosis markers were also analyzed in PCGEM-downregulate cells. The results indicated that the level Bax was enhanced but the level of Bcl-2 was depleted (Fig. S1C). Overall, PCGEM1 was highly expressed in NSCLC cells and knockdown of it suppressed NSCLC cell growth.

### PCGEM1 acts as a miR-433-3p sponge in NSCLC

**Fig. 2:**
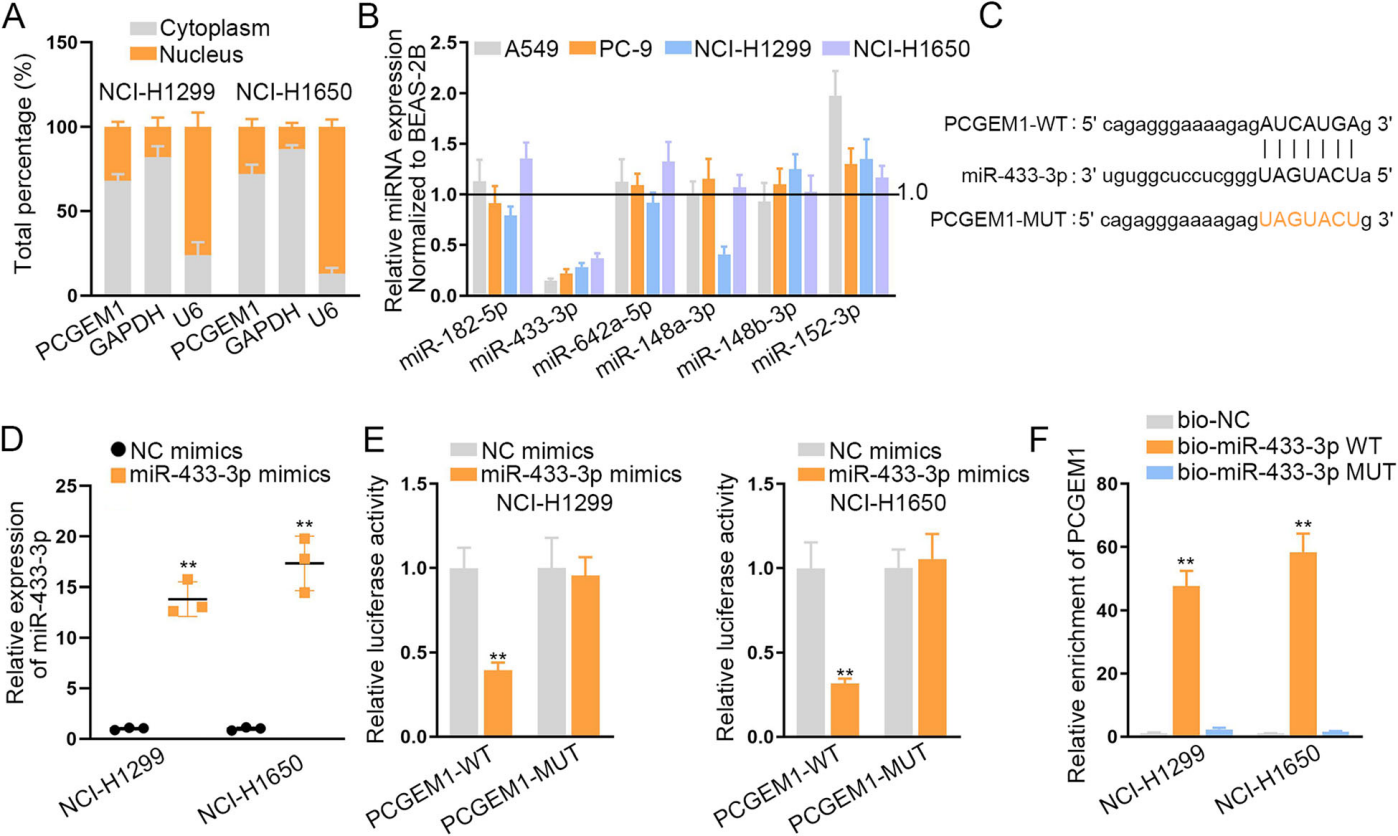
PCGEM1 serve as a miR-433-3p sponge in NSCLC. **a** The distribution of PCGEM1 in NCI-H1299 and NCI-H1650 cells was tested through subcellular fractionation assay. **b** The expressions of six miRNAs were tested through qRT-PCR in NSCLC cell lines (A549, PC-9, NCI-H1299 and NCI-H1650) and the human lung bronchial epithelial cell line (BEAS-2B). **c** The binding site of PCGEM1 and miR-433-3p was predicted by ENCORI database. **d** The utilization of qRT-PCR was utilized to test the overexpression efficiency of PCGEM1 in NCI-H1299 and NCI-H1650 cells. **e** The luciferase report experiments were utilized to confirm the binding situation of PCGEM1 and miR-433-3p in NCI-H1299 and NCI-H1650 cells. **f** RNA pull down assays were conducted to verify the interaction of PCGEM1 and miR-433-3p in NCI-H1299 and NCI-H1650 cells. ^**^P < 0.01

After investigating the functions of PCGEM1 in NSCLC cells, we began to search the regulatory mechanisms of PCGEM1. Firstly, we conducted subcellular fractionation assay to identify the location of PCGEM1 in NCI-H1299 and NCI-H1650 cells (Fig. 2a). The outcomes indicated that PCGEM1 was mostly distributed in the cytoplasm, suggesting the potential regulation at post-transcriptional level. Thus, we conjectured that PCGEM1 could act as ceRNA to sponge miRNAs and regulate mRNAs at post-transcriptional level. Accordingly, we discovered six miRNAs (miR-182-5p, miR-433-3p, miR-642a-5p, miR-148a-3p, miR-148b-3p and miR-152-3p) that might combine with PCGEM1 through the prediction of ENCORI (http://starbase.sysu.edu.cn/index.php) database. Then, we carried out qRT-PCR to test their expressions to select the most appropriate miRNAs (Fig. 2b). The results revealed that miR-433-3p expression was lower than that of other miRNAs in NSCLC cell lines (A549, PC-9, NCI-H1299 and NCI-H1650) normalized to the human lung bronchial epithelial cell line (BEAS-2B). Furthermore, these six miRNAs were also subjected to QRT-PCR analysis in 50 pairs of NSCLC tissues and adjacent normal tissues. According to the results, only miR-433-3p presented low level in NSCLC tissues (Fig. S2A). We also detected the level of miR-433-3p in normal BEAS-2B cell compared to four NSCLC cells. It was found that miR-433-3p was expressed higher in BEAS-2B cell compared to all four NSCLC cells (Fig. S2B). So we selected miR-433-3p to perform the following assays and detect the correlation of miR-433-3p and PCGEM1. In accordance with the ENCORI database, we discovered the binding sites of miR-433-3p and PCGEM1 (Fig. 2c). After we overexpressed miR-433-3p in NCI-H1299 and NCI-H1650 cells by transfecting with miR-433-3p mimics, we carried out the luciferase reporter assays to prove the correlation of miR-433-3p and PCGEM1 (Fig. 2d, e). As expected, overexpressed miR-433-3p inhibited the luciferase activity of PCGEM1-WT, but didn’t work for PCGEM1-MUT, indicating that PCGEM1 could bind to miR-433-3p. Subsequently, RNA pull down experiments were adopted to test their interaction (Fig. 2f). Visibly, the enrichment of PCGEM1 was effectively ascended by biotinylated miR-433-3p-WT. These experiments indicated that miR-433-3p could interact with PCGEM1. Taken together, PCGEM1 can act as a sponge of miR-433-3p in NSCLC.

### WTAP is a downstream target gene of miR-433-3p in NSCLC

**Fig. 3:**
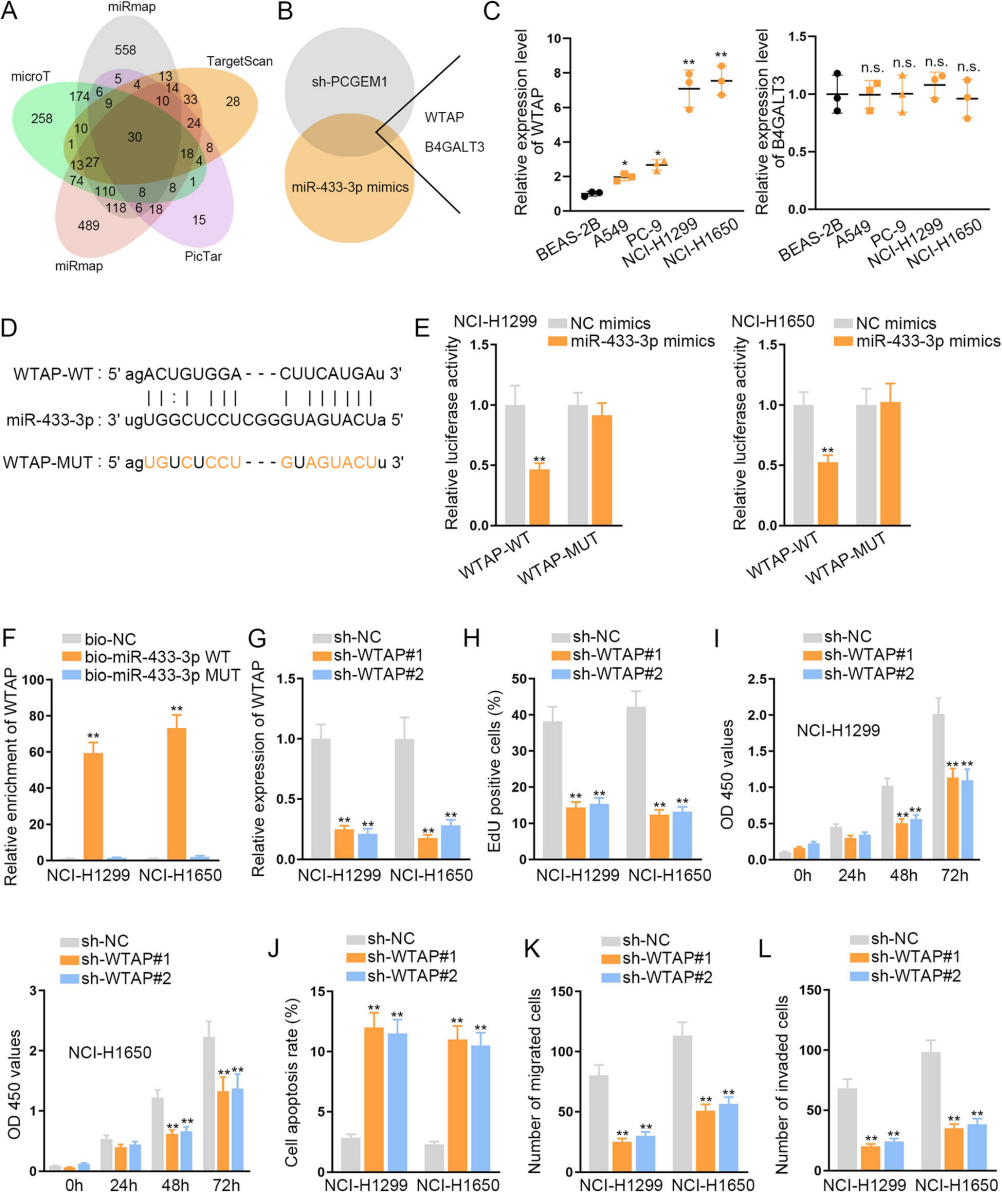
WTAP is a downstream target gene of miR-433-3p in NSCLC. **a** ENCORI was adopted to predict the possible mRNAs for miR-433-3p. **b** qRT-PCR was carried to screen out the mRNAs which could be declined by silenced PCGEM1 and overexpressed miR-433-3p. **c** qRT-PCR continued to test the expressions of WTAP and B4GALT3 in NSCLC cell lines (A549, PC-9, NCI-H1299 and NCI-H1650) and the human lung bronchial epithelial cell line (BEAS-2B). **d** The binding site of WTAP and miR-433-3p was predicted by ENCORI. **e** The binding relationship of WTAP and miR-433-3p was confirmed through luciferase report experiments. **f** RNA pull down assays were applied to verify the interaction of WTAP and miR-433-3p in NCI-H1299 and NCI-H1650 cells. **g** The knockdown efficiency of WTAP was tested by qRT-PCR in NCI-H1299 and NCI-H1650 cells. **h**, **i** Cell proliferation ability was evaluated through EdU and CCK-8 experiments after WTAP was knocked down in NCI-H1299 and NCI-H1650 cells. **j** Cell apoptosis was measured in NSCLC cells through flow cytometry when WTAP was silenced in NCI-H1299 and NCI-H1650 cells. **k**, **l** Transwell assays were conducted to estimate cell migration and invasion in cells which were transfected with silenced WTAP in NCI-H1299 and NCI-H1650 cells. ^*^P < 0.05, ^**^P < 0.01, n.s.: no significance

For further investigation on the regulatory mechanism of PCGEM1/miR-433-3p axis in NSCLC, we found out the possible target genes of miR-433-3p through ENCORI database. Under the prediction of miRmap, microT, miRanda, PicTar and TargetScan, thirty mRNAs were discovered (Fig. 3a). Then we utilized the qRT-PCR to screen out the most possible mRNAs which could be regulated concurrently by both silenced PCGEM1 and overexpressed miR-433-3p (Fig. 3b). According to the results, WTAP and B4GALT3 were picked out. Hence, we further conducted qRT-PCR to test the expression of WTAP and B4GALT3 in NSCLC cell lines (A549, PC-9, NCI-H1299 and NCI-H1650) and the human lung bronchial epithelial cell line (BEAS-2B) (Fig. 3c). The outcomes displayed that WT1 associated protein (WTAP) exhibited a high expression level in NSCLC cells while B4GALT3 expression was not notable. Consistently, we observed that WTAP was expressed at a relative high level in NSCLC cells compared to BEAS-2B cell (Fig. S2C). Following, we selected WTAP to conduct the later experiments. And then we discovered the binding sites of WTAP and miR-433-3p through ENCORI (Fig. 3d). Luciferase reporter experiments unmasked that the luciferase activity of WTAP-WT was restrained by the up-regulation of miR-433-3p, while that of WTAP-MUT was affected (Fig. 3e). RNA pull down assay indicated that WTAP was enriched in bio-miR-433-3p-WT group rather than bio-miR-433-3p-MUT and bio-NC groups (Fig. 3f). Thus, we proved that WTAP served as the target of miR-433-3p. But the functions of WTAP in NSCLC cells were still unclear. Hence, we carried out several functional experiments. Firstly, we detected the knockdown efficiency of WTAP in NCI-H1299 and NCI-H1650 cells and the results indicated that WTAP expression was effectively declined after we transfected sh-WTAP into NCI-H1299 and NCI-H1650 cells (Fig. 3g). According to the results of EdU and CCK-8 assays, we discovered that the proliferative capability of NCI-H1299 and NCI-H1650 cells was suppressed when WTAP was inhibited (Fig. 3h, i). Flow cytometry analysis indicated that cell apoptosis could be expedited by the down-regulation of WTAP (Fig. 3j). Moreover, cell migration and invasion were also hampered due to the lack of WTAP (Fig. 3k, l). In short, WTAP is a downstream target gene of miR-433-3p in NSCLC.

### PCGEM1 promotes NSCLC progression via miR-433-3p/WTAP axis

**Fig. 4:**
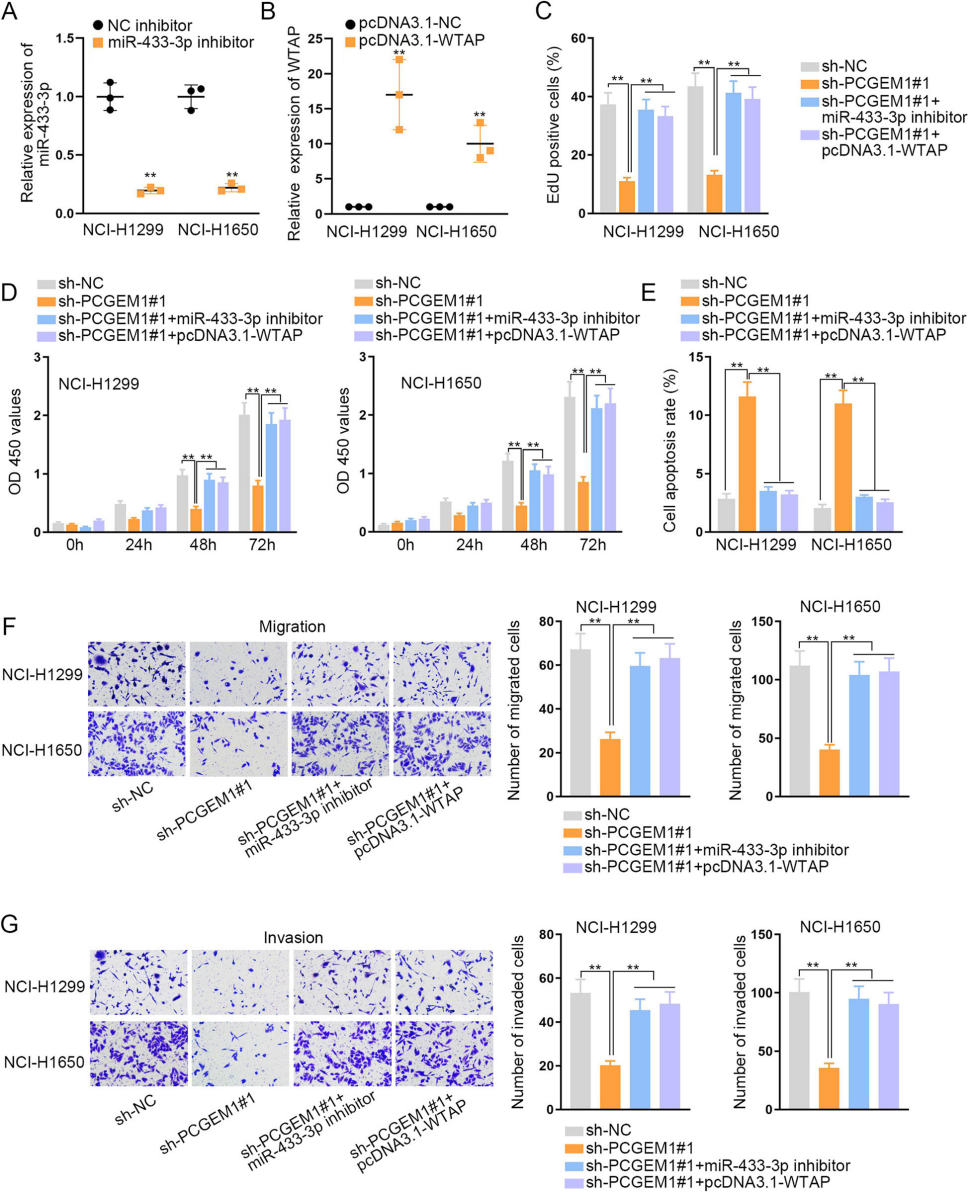
PCGEM1 promotes NSCLC progression via miR-433-3p/WTAP axis. **a**, **b** The interference efficiency of miR-433-3p and the overexpression efficiency of WTAP were tested through qRT-PCR in NCI-H1299 and NCI-H1650 cells which transfected with miR-433-3p inhibitor or pcDNA3.1-WTAP. **c**, **d** EdU and CCK-8 experiments were utilized to measure the influence of miR-433-3p knockdown or WTAP overexpression on the inhibited cell proliferation which caused by PCGEM1 depletion. **e** The cell apoptosis ability which was restrained by PCGEM1 depletion was tested when miR-433-3p was inhibited or WTAP was up-regulated through flow cytometry analysis. **f**, **g** Cell migration and invasion inhibited by silenced PCGEM1 were evaluated after knockdown of miR-433-3p or overexpression of WTAP in NCI-H1299 and NCI-H1650 cells. ^**^P < 0.01

In the end, we carried out the rescue experiments to prove the regulatory mechanism of PCGEM1 in NSCLC. We silenced miR-433-3p and overexpressed WTAP in NCI-H1299 and NCI-H1650 cells separately and tested the transfection efficiency through qRT-PCR (Fig. 4a, b). We could visibly observe that the expression of miR-433-3p was declined and the expression of WTAP was increased. Then, EdU staining and CCK-8 experiments were conducted and we discovered that the inhibited cell proliferation which was caused by silenced PCGEM1 was fully accelerated by knockdown of miR-433-3p or up-regulation of WTAP (Fig. 4c, d). And it was indicated by flow cytometry analysis that cell apoptotic ability could be expedited when miR-433-3p was inhibited or PCGEM1 was overexpressed (Fig. 4e). In addition, through transwell experiments, we found that the lack of miR-433-3p or up-regulation of WTAP could offset the inhibitive function of knocking down PCGEM1 on cell migration and invasion (Fig. 4f, g). All of these experiments indicated that PCGEM1 accelerates NSCLC progression via miR-433-3p/WTAP axis.

## Discussion

The incidence and mortality of NSCLC are increasing each year in the global scale. Despite researchers are trying to probe more effective treatment, the five-year survival rate of patients with NSCLC is not optimistic. In recent years, lncRNAs have been discovered to be singularly expressed in multifarious diseases, including cancers. They were considered to be associated with the occurrence and development of human cancers [8]. Considerable number of cancers has been discovered to be closely related to lncRNAs, including NSCLC [18]. For example, linc00673 can regulate cell proliferation of NSCLC through sponging miR-150-5p [19]. MAFG-AS1 facilitates cell migration and invasion in NSCLC by sponging miR-339-5p [20]. PCGEM1 is an lncRNA and it has been reported to facilitate the development of some cancers, such as prostate cancer [12], endometrial carcinoma [13] and ovarian carcinoma [14]. Nevertheless, the functions of PCGEM1 in NSCLC still need to be explored. In our research, we examined the expression of PCGEM1 in NSCLC cells firstly and we discovered a strong and anomalous expression level of PCGEM1. Then, we took a series of functional experiments to detect the specific functions of PCGEM1. And we found that knockdown of PCGEM1 retrained cell proliferation, migration and invasion in NSCLC cells. At the same time, it also accelerated cell apoptosis. These experiments were all indicated that PCGEM1 served as an oncogene in NSCLC.

TP53 can involve in cancer progression. Here, we analyzed whether p53 participated in PCGEM1-mediated NSCLC cellular processes. At first, we detected whether p53 induced the transcriptional activation of PCGEM1. As a result, we determined that p53 could not affect PCGEM1 transcription. Furthermore, we examined the protein levels of p53 and apoptotic markers. Intriguingly, PCGEM1 negatively regulated p53 and suppressed apoptotic pathway. Therefore, PCGEM1 regulated apoptotic pathway possibly depend on p53.

Subsequently, we investigated the downstream mechanism of PCGEM1 in NSCLC. Recently, an increasing number of researches indicate that lncRNA could serve as ceRNA to regulate mRNAs expressions through sponging miRNAs [15]. CeRNA network was a kind of regulatory mechanism after transcription [21]. Lots of lncRNAs have been proven to serve as ceRNAs to take part in the processes of cancers. For example, PTENP1 serves as ceRNA to control PTEN level through sponging miR-106b and miR-93 in gastric cancer [22]. And PDL1 acts as ceRNAs in triple negative breast cancer via regulating miR-34a [23]. In our research, we discovered that the main distribution of PCGEM1 was in cytoplasm. Then we sought out the possible miRNAs. Assorted reports indicate that miRNAs are also crucial for biological process and they can combine with lncRNAs to regulate cancer development. Here, miR-433-3p was proven to be the downstream miRNA of PCGEM1. According to a previous study, PCGEM1 can interact with miR-433-3p to modulate renal carcinoma progression [24]. Nevertheless, whether PCGEM1 exerted functions in NSCLC through miR-433-3p remain unclear. In our current study, we determined the interaction between PCGEM1 and miR-433-3p in NSCLC for the first time.

WTAP was discovered to exert carcinogenic effect in several cancers. For example, WTAP expedite cell migration and invasion in cholangiocarcinoma [25]. WTAP is highly expressed in bladder cancer cells and overexpression of it indicates poor prognosis [26]. To our knowledge, the functions of WTAP in NSCLC remain undefined. In our research, we discovered that the expression of WTAP was extremely high in NSCLC cells. Through functional experiments, we found that silenced WTAP could restrain cell proliferative ability. Mechanistically, we verified that WTAP was the target of miR-433-3p, unveiling a novel miR-433-3p/WTAP axis in NSCLC. More importantly, WTAP could recover the inhibitory function of PCGEM1 silencing on NSCLC progression, suggesting that PCGEM1 exerted oncogenic functions in NSCLC through serving as a ceRNA to modulate miR-433-3p/WTAP axis.

To sum up, we discovered that highly expressed PCGEM1 could accelerate NSCLC progression via miR-433-3p/WTAP axis, offering a new idea for NSCLC treatment. This study unveiled a novel ceRNA pathway in NSCLC progression. Nevertheless, lack of the deep investigation on the multiple targets of PCGEM1 is a limitation of the current study. We will explore whether PEGEM1 exert functions through regulating multiple targets in our future study. Moreover, the specific mechanism by which PCGEM1 modulated p53 protein.

## Conclusions

PCGEM1 is highly expressed in NSCLC cells. Highly expressed PCGEM1 accelerates NSCLC progression via miR-433-3p/WTAP axis, offering a new idea for curing NSCLC.

## Supplementary information

**Additional file 1: Figure S1.** (A) PCGEM1 expression was tested in NSCLC tissues and corresponding normal tissues by QRT-PCR. (B) Luciferase reporter assay was conducted to analyze the transcriptional activity of PCGEM1 under the ectopic expression of p53. (C) The levels of p53 and apoptotic markers (Bax and Bcl-2) were tested in PCGEM1-silenced NSCLC cells by western blot. ^**^P < 0.01. n.s indicated data were not statistically significant.

**Additional file 2: Figure S2.** (A) The expression patterns of six candidate miRNAs in paired NSCLC and non-tumor tissues were detected by qRT-PCR. (B) The level of miR-433-3p was determined by qRT-PCR in BEAS-2B cells by comparing to four NSCLC cells, respectively. (C) WTAP expression in 50 NSCLC tissues and corresponding normal tissues was identified through qRT-PCR. ^**^P < 0.01. n.s indicated data were not statistically significant.

**Additional file 3.**

## Data Availability

Research data and materials are available from the corresponding author on reasonable request.

## Abbreviations

NSCLC: Non-small cell lung cancer
PCGEM1: PCGEM1 prostate-specific transcript
WTAP: WT1 associated protein
lncRNAs: Long noncoding RNAs
mRNAs: Messenger RNAs
miRNAs: MicroRNAs
ceRNA: Competing endogenous RNA
ATCC: American Type Culture Collection
BEGM: Bronchial Epithelial Cell Growth Medium
RPMI: Roswell Park Memorial Institute
FBS: Fatal Bovine Serum
QRT-PCR: Real-time quantitative polymerase chain reaction
NC: Negative control
CCK-8: Cell Counting Kit-8
EdU: 5-ethynyl-2′-deoxyuridine
DAPI: 4′,6-diamidino-2-phenylindole
WT: Wild-type
Mut: Mutant
SD: Standard deviation
ANOVA: Analysis of Variance
