# Supplementary figures and images for: LncRNA PCGEM1 accelerates non-small cell lung cancer progression via sponging miR-433-3p to upregulate WTAP
Source: BMC Pulm Med. 2020 Aug 12;20:213. doi: 10.1186/s12890-020-01240-5 (PMC7425603; doi:10.1186/s12890-020-01240-5)

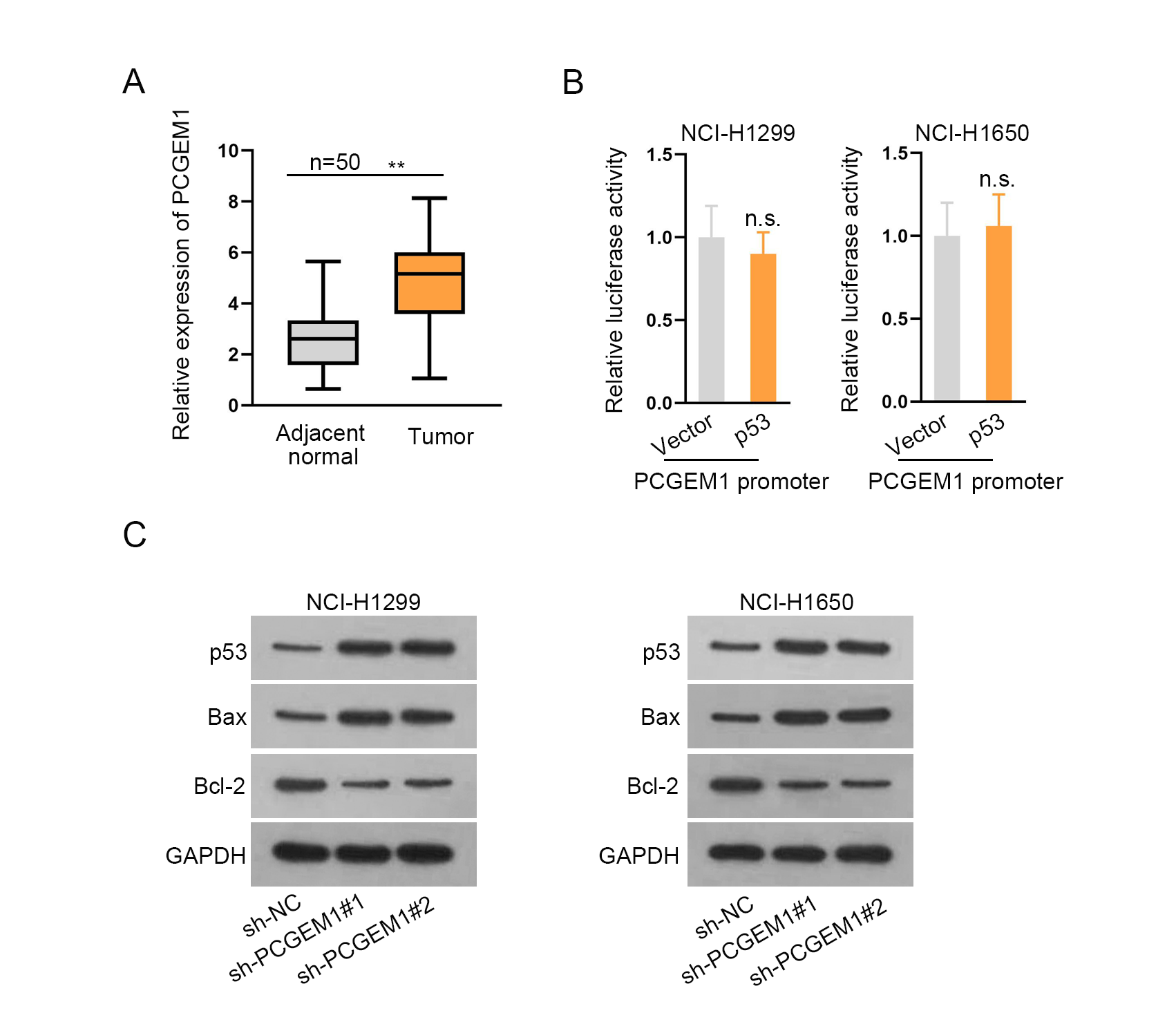

Supplement: Supplementary file 1 — Additional file 1: Figure S1. (A) PCGEM1 expression was tested in NSCLC tissues and corresponding normal tissues by QRT-PCR. (B) Luciferase reporter assay was conducted to analyze the transcriptional activity of PCGEM1 under the ectopic expression of p53. (C) The levels of p53 and apoptotic markers (Bax and Bcl-2) were tested in PCGEM1-silenced NSCLC cells by western blot. **P < 0.01. n.s indicated data were not statistically significant. [file 12890_2020_1240_MOESM1_ESM.tif]

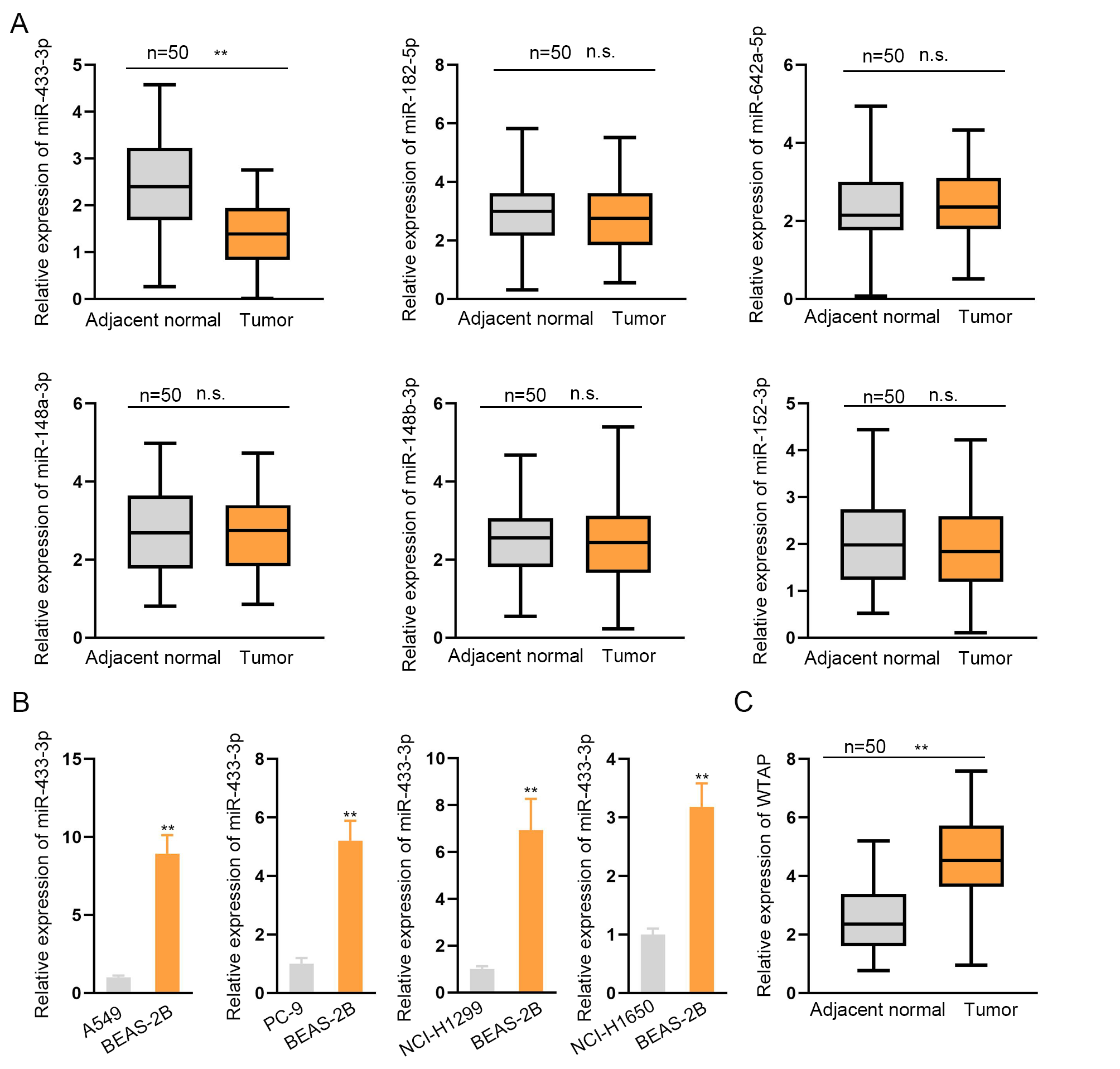

Supplement: Supplementary file 2 — Additional file 2: Figure S2. (A) The expression patterns of six candidate miRNAs in paired NSCLC and non-tumor tissues were detected by qRT-PCR. (B) The level of miR-433-3p was determined by qRT-PCR in BEAS-2B cells by comparing to four NSCLC cells, respectively. (C) WTAP expression in 50 NSCLC tissues and corresponding normal tissues was identified through qRT-PCR. **P < 0.01. n.s indicated data were not statistically significant. [file 12890_2020_1240_MOESM2_ESM.tif]

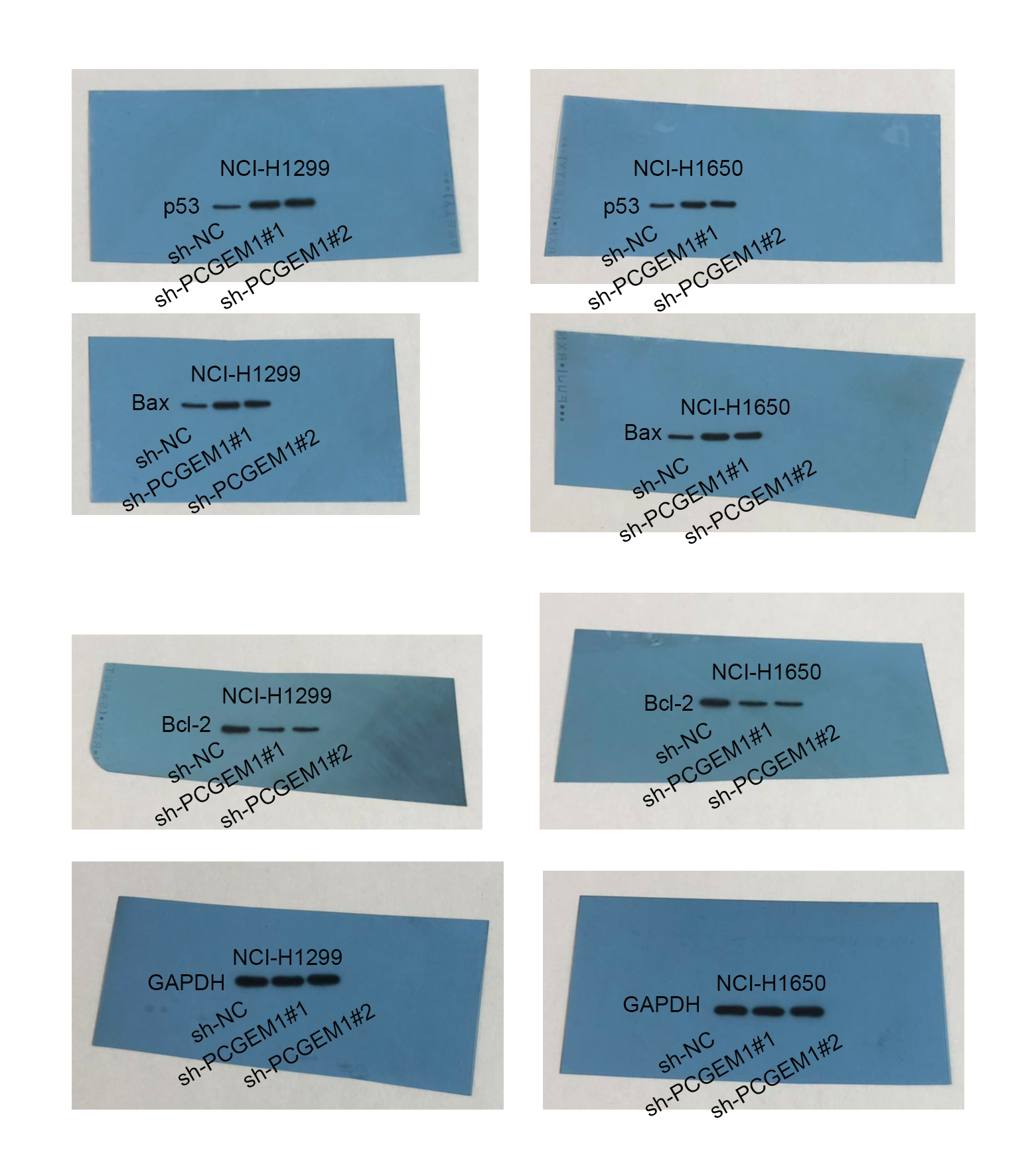

Supplement: Supplementary file 3 — Additional file 3. [file 12890_2020_1240_MOESM3_ESM.tif]
